# Magnetic Soft Microrobot Design for Cell Grasping and Transportation

**DOI:** 10.34133/cbsystems.0109

**Published:** 2024-04-25

**Authors:** Fanghao Wang, Youchao Zhang, Daoyuan Jin, Zhongliang Jiang, Yaqian Liu, Alois Knoll, Huanyu Jiang, Yibin Ying, Mingchuan Zhou

**Affiliations:** ^1^ College of Biosystems Engineering and Food Science, Zhejiang University, Hangzhou 310058, China.; ^2^ TUM School of Computation, Information, and Technology, Garching 85748, Germany.

## Abstract

Manipulating cells at a small scale is widely acknowledged as a complex and challenging task, especially when it comes to cell grasping and transportation. Various precise methods have been developed to remotely control the movement of microrobots. However, the manipulation of micro-objects necessitates the use of end-effectors. This paper presents a study on the control of movement and grasping operations of a magnetic microrobot, utilizing only 3 pairs of electromagnetic coils. A specially designed microgripper is employed on the microrobot for efficient cell grasping and transportation. To ensure precise grasping, a bending deformation model of the microgripper is formulated and subsequently validated. To achieve precise and reliable transportation of cells to specific positions, an approach that combines an extended Kalman filter with a model predictive control method is adopted to accomplish the trajectory tracking task. Through experiments, we observe that by applying the proposed control strategy, the mean absolute error of path tracking is found to be less than 0.155 mm. Remarkably, this value accounts for only 1.55% of the microrobot’s size, demonstrating the efficacy and accuracy of our control strategy. Furthermore, an experiment involving the grasping and transportation of a zebrafish embryonic cell (diameter: 800 μm) is successfully conducted. The results of this experiment not only validate the precision and effectiveness of the proposed microrobot and its associated models but also highlight its tremendous potential for cell manipulation in vitro and in vivo.

## Introduction

Cells serve as the basic unit of organism structure and function, as well as the most basic living system [1]. Cell manipulation is a fundamental aspect of cell research, and advancements in single-cell sorting and trapping technology have presented challenges and opportunities in various fields such as biological engineering, medical engineering, agricultural engineering, chemical engineering, etc [2–6].

Cells are commonly manipulated using micro- or nano-sized tools, including pillars, membranes, fibers, and electrodes. Several researchers have developed various methods for wireless actuation, such as optical, acoustic, microfluidic, magnetic, and others, to address these tasks [7–12]. Xie et al. [13] utilized optical tweezers to control the orientation of a single cell before cell surgery, which greatly improved the surgery efficiency. Ren et al. [14] proposed a microbubble-based microrobot capable of controllable high-speed swimming and moving through obstacles. Schmidt et al. [15] proposed a magnetic microhelix to capture an immotile sperm and deliver it to the oocyte for fertilization. Among them, electromagnetic actuation has the advantages of precision, great power, good biological compatibility, and the ability to be remotely controlled [16–21].

To manipulate cells through magnetic microrobots, lots of studies have been investigated with different system designs. In [22], Ahmad et al. proposed a mobile microgripper actuated using laser optothermal and magnetic actuation in liquid environments. The position of the microgripper is controlled by the magnetic platform and the gripping motion is actuated by the optothermal platform. So the control complexity is reduced by full decoupling. However, this method introduces 2 actuating sources, resulting in high system complexity. Zhang et al. [23] proposed a reliable mobile magnetic microgripper to grasp and transfer micro-objects. Although only 1 single global magnetic field is applied, the 3 variables of the microgripper, i.e., its shape, orientation, and position, are still controlled independently. However, the proposed microgripper is composed of several rigid bodies that are destructive for biological application. Compared to manipulating micro-objects using a rigid gripper, a soft gripper is more suitable for reducing the damage in cells. Huang et al. [24] proposed a multimode quadruped microrobot which is composed of 4 magnetic soft thin-film legs and a nonmagnetic body. The bionic microrobot can grasp, transport, and release microbeads and shows great potential for manipulation in vivo. However, the locomotion of the microrobot requires complex magnetic field input that needs to change the direction of the current frequently and may cause low control precision. In addition, none of these systems operate cells. To simplify the manipulating system, reduce the damage to cells, and maintain good control accuracy, a system for cell grasping and transportation is designed.

In this paper, a specific microrobot is designed and fabricated to realize the grasping and transportation simultaneously with a solo magnetic actuator. A magnetic platform with a minimum number of coils is built up for control of the proposed microrobot. The grasping model of the soft gripper and the kinematic model of the microrobot are built. The model predictive control (MPC) and extended Kalman filter (EKF) are adopted to realize robust trajectory tracking. The cell grasping and transportation experiment shows that the proposed system has the advantages of low complexity, high precision, and good biocompatibility, which lays great potential for cell manipulation. The main contributions of this paper are summarized as follows:

1. A magnetic control platform and a soft microrobot are designed that allow for minimal inputs to simultaneously control the rotation, movement, and grasping of the microrobot. This integrated system provides a novel solution for precise micromanipulation tasks.

2. The EKF-MPC algorithm is designed to track trajectories that achieve accurate trajectory tracking with a mean absolute error (MAE) of less than 0.155 mm, and a bending deformation model is proposed and verified to grasp the target.

3. Utilizing our system, precise and automated point-to-point transportation of zebrafish cells is achieved with a releasing MAE of 0.067 mm. This capability demonstrates the high accuracy and automation potential of our proposed approach in cell manipulation tasks.

## Materials and Methods

The following section outlines the design of the microrobot and the construction of the electromagnetic system. A bending deformation model for the microgripper is established. A motion control model is built for navigating the microrobot. Based on these models, a grasping and releasing module is proposed to control the closing and opening of the microgripper, while an EKF-MPC trajectory control method is designed to track the reference path.

### Microrobot fabrication and system design

**Fig. 1.**
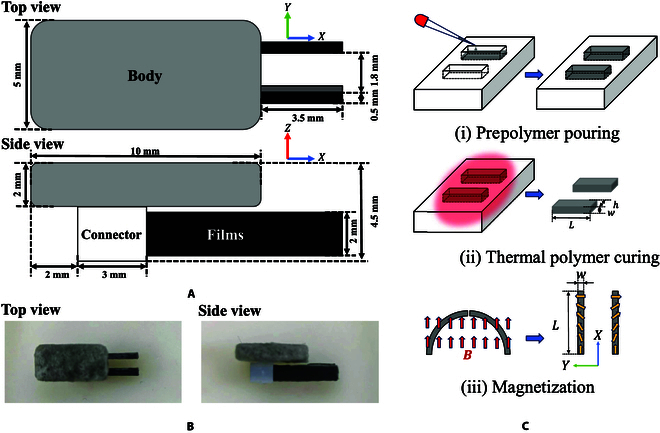
The design and the fabrication process of the microrobot. (A) The microrobot is composed of 2 magnetic films, a nonmagnetic connector, and a nonmagnetic body. (B) The image of the microrobot in the real world. (C) The fabrication process of the microgripper.

The proposed microrobot is composed of a nonmagnetic body (polyvinyl chloride), a 3-dimensional-printed connector (resin), and 2 magnetic films as shown in Fig. 1A and B where *L*, *w*, and *h* represent the length, width, and height of the film. The films used on the microrobot have dimensions of *L* = 10 mm, *w* = 0.5 mm, and *h* = 2 mm. The base material of the film is an Ecoflex 00-10 polymer matrix (Smooth on Inc.; density: 1.04 g/cm^3^) loaded with neodymium-iron-boron (NdFeB) magnetic microparticles (MQP-15-7, Magnequench; average diameter: 5 μm, density: 7.61 g/cm^3^) according to a mass ratio of 1:1 (mass of NdFeB microparticles to the mass of Ecoflex-10) [25]. To get a user-defined shaped film, a mold (polytetrafluoroethylene) is used to fit the cuboid shape. In this study, the fabrication process of these films is presented in Fig. 1C. Firstly, the prepolymer is cast into the mold with cubic space. Next, they are put into the oven to perform thermal polymer curing. Finally, the films are bent to a one-fourth circular shape on a circular mold and magnetized under 1.5 T. Due to the symmetry of their magnetization and the principle that magnets with the same magnetic pole repel one another, a misalignment on the z-axis will appear when their free endpoints are close enough. To deal with this problem, a nonmagnetic cuboid (polydimethylsiloxane) is attached to the right film, which enlarges the distance between the 2 films when grasping. These 2 films form the microgripper for grasping and releasing.

**Fig. 2.**
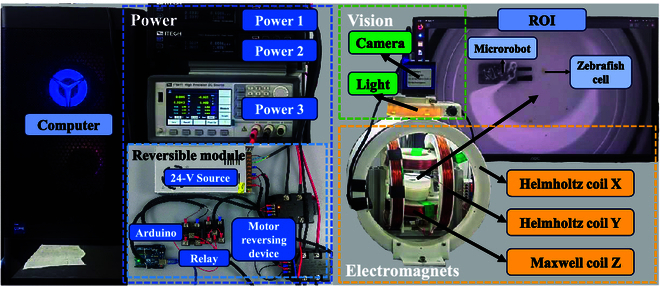
The schematic diagram of the magnetic platform. The microrobot is placed at the center of 2 pairs of Helmholtz coils and 1 pair of Maxwell coils arranged orthogonally. Three dc powers are supplied to exert current on coils to produce the magnetic field. The top-view camera is equipped with a light source to provide stable and reliable images. A computer is adopted to control this system.

To propel the microrobot and the microgripper using the only actuating source, a magnetic platform with minimum inputs is designed where 2 Helmholtz coils are placed orthogonal in the x-y plane to produce a uniform magnetic field and 1 Maxwell coil is equipped in the z-axis to produce a uniform gradient magnetic field. To fully control the microrobot with minimal inputs, 3 independent coils are needed to drive the microrobot’s 3 outputs: rotation, movement, and grasping. The magnetic platform as shown in Fig. 2 can rotate the magnetic microrobot in any direction and apply a driving force in the direction of its current magnetic moment [26]. To design this magnetic platform for actuating the microrobot, a prototype of the microrobot is first made and used to estimate the magnetic demand for grasping and movement. The estimated magnetic properties are used to design the 3 coils. After manufacturing the coils, the actual parameters are measured and listed in Table 1, where *D_i_*, *D_o_*, *T*, *N*, and *R* represent the inner diameter of the coils, the outer diameter of the coils, the thickness of the coils in the axial direction, the number of turns, and the resistance, respectively.

**Table 1.** The properties of 3 pairs of coils

Coils	Axis	*D_i_* (mm)	*D_o_* (mm)	*T* (mm)	*N*	*R* (Ω)	Magnetic characteristic
Helmholtz	X	190	220	15	225	9.80	2.0788 mT/A
	Y	140	160	15	150	4.86	1.7311 mT/A
Maxwell	Z	70	100	15	225	4.90	0.0515 T/(m⋅A)

The microrobot and the zebrafish cell are placed in a container in the center of the magnetic platform as shown in the region of interest in Fig. 2. To induce the magnetic field for actuating the microrobot, a power module is built up to supply bidirectional current on the 3 coils. The power module is composed of 2 direct-current (dc) programmable power sources (IT-M3432, ITECH, China) for Helmholtz coils that are capable of providing 60 V, 30-A current, and 1 (IT6411, ITECH, China) for Maxwell coil that is capable of providing ±9-V, 5-A current. To facilitate the application of bidirectional current on Helmholtz coils, a reversible module has been constructed within the power module. This reversible module includes a 24-V power source, an Arduino, 2 relays, and 2 motor reversing devices. The positive and negative current switching frequencies of IT-M3432 with a reversible module and IT6411 are approximately 20 and 15 Hz, respectively. These frequencies are deemed satisfactory for the proper functioning of the system. To provide stable visual feedback, a top-view camera (HY-1135, HAYEAR, China) equipped with a light source is assembled. The image acquired from the camera is 1, 920 × 1, 080 pixels with an effective pixel size of 24 μm. The overall system is implemented through Python in robot operation system by a computer with an Intel Core 7 Duo 3.8-GHz processor and a 12-GB memory 3060 GPU running on Ubuntu 20.04.

### Microrobot modeling and control

**Fig. 3.**
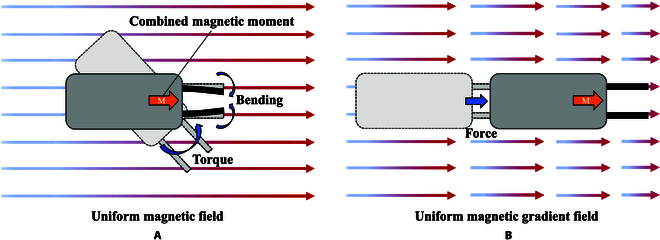
The diagram of the motion model. (A) The microrobot rotates and the microgripper closes under the uniform magnetic field. (B) The microrobot moves in the uniform magnetic gradient field.

The benefit of the proposed design is that we can achieve grasping control and motion control simultaneously with the solo magnetic actuation source. The microgripper is actuated by the magnitude of the uniform magnetic field to close and open the soft films to grasp and release the target cell. The velocity of the microrobot is controlled by the magnitude of the gradient magnetic field, and the orientation is controlled by the direction of the uniform magnetic field. Figure 3 illustrates the motion model of the microrobot.

#### Microgripper modeling

**Fig. 4.**
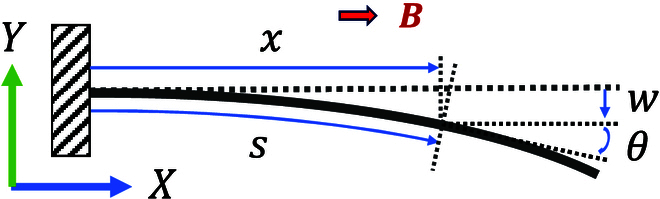
The geometric definition of the simplified cantilever beam in the static state. The film is placed under a uniform magnetic field on the x-axis.

In this paper, a magnetic microgripper is shape-programmed and used for both movement and grasping. Because the 2 films are symmetric about the x-axis as shown in Fig. 1C, the combined magnetic moment is always along the x-axis. To simplify this model, the influence of the internal magnetic force on a single film and the mutual influence between the 2 films are ignored. Because of the symmetry of the 2 films, the left film is analyzed and its magnetization is described as:

$$m\left(s\right)=\left[\begin{array}{c} m_{x}\\ m_{y}\\ m_{z} \end{array}\right]=\bar{m}\;\left[\begin{array}{c} \text{cos}\left(\frac{\pi }{2L}s\right)\\ \text{sin}\left(\frac{\pi }{2L}s\right)\\ 0 \end{array}\right]$$ (1)

where $\bar{m}$ and *s* represent the magnetization and the length along the film, respectively. The direction of the combined magnetic moment of the microgripper is always aligned with the uniform magnetic field. So the direction of the uniform magnetic field in the robotic frame is always in the x-axis. To model the bending properties of the microgripper, the film is treated as a cantilever beam. Based on the above simplifications, the film in the static state is shown in Fig. 4, where *x*, *w*, and *θ* denote the position in the x-axis, the deflection in the y-axis, and the bending angle, respectively. The bending angle *θ* is defined as the counterclockwise angle between the tangent line of the curved beam and the positive x-axis. Take the beam at *s* = *s*_0_ as the breakpoint and analyze the bending moment *M*(*s*_0_) of the right remaining beam:

$$M\left(s_{0}\right)=\int_{s_{0}}^{L}\;\tau \left(s\right)\mathrm{Ads}$$ (2)

where *A* = *wh* denotes the cross-sectional area. *τ* denotes the magnetic torque per unit volume and is calculated as follows:

$$\begin{array}{c} \begin{array}{l} \tau \left(s\right)=\left[0\;0\;1\right]R\left(s\right)m\left(s\right)\times B\\ \;=\left[0\;0\;1\right]\left[\begin{array}{ccc} \text{cos}\left(\theta \left(s\right)\right) & -\text{sin}\left(\theta \left(s\right)\right) & 0\\ \text{sin}\left(\theta \left(s\right)\right) & \text{cos}\left(\theta \left(s\right)\right) & 0\\ 0 & 0 & 1 \end{array}\right]\left[\begin{array}{c} \text{cos}\left(\frac{\pi }{2L}s\right)\\ \text{sin}\left(\frac{\pi }{2L}s\right)\\ 0 \end{array}\right]\times \left[\begin{array}{c} B\\ 0\\ 0 \end{array}\right]\\ \;=-B\;\text{sin}\left(\theta \left(s\right)+\frac{\pi }{2L}s\right) \end{array} \end{array}$$ (3)

where ***R*** and ***B*** represent the rotational matrix and the uniform magnetic field, respectively. Assume *θ* = *f*(*s*), so it is obvious that *f*(0) = 0 in this cantilever beam. The shear force in the beam is neglected, so the beam is treated as a pure bending beam, then:

$$M\left(s\right)=\frac{\mathrm{EI}}{\rho }=\mathrm{EI}\frac{\mathrm{d\theta}}{\mathrm{ds}}$$ (4)

Substitute Eqs. 3 and 4 into Eq. 2:

$$\begin{array}{c} {\left.M\left(s_{0}\right)=-\mathrm{AB}\int_{s_{0}}^{L}\;\text{sin}\left(f\left(s\right)+\frac{\pi }{2L}s\right)\mathrm{ds}=\mathrm{EI}\frac{\mathrm{d\theta}}{\mathrm{ds}}\right|}_{s=s_{0}}=\mathrm{EI}f\prime \left(s_{0}\right) \end{array}$$ (5)

where *E* and $I=\frac{{\mathrm{hw}}^{3}}{12}$ denote the Young’s modulus and the second moment of area, respectively. It is easy to derive that f′(*L*) = 0 from the above equation. At the same time, the above equation satisfies for all *s*_0_ ∈ [0, *L*]. So derivative *s*_0_ at both sides:

$$\begin{array}{cc} f^{\prime \prime }\left(s_{0}\right) & =-\frac{\mathrm{AB}}{\mathrm{EI}}\text{sin}\left(f\left(s_{0}\right)+\frac{\pi }{2L}s_{0}\right)\\ s.t.f\left(0\right) & =0;f\prime \left(L\right)=0;s_{0}\in [0,L] \end{array}$$ (6)

So far, the bending deformation differential model of the microgripper has been constructed.

#### Microrobot motion model

In general, a Maxwell coil generates a uniform gradient magnetic field not only in the axial direction but also in the radian plane. The gradient field in 3-dimensional induced by a z-axis Maxwell coil is described [26] as:

$$\begin{array}{c} \nabla B=\left[\begin{array}{ccc} -0.5\tilde{G_{z}}I_{z} & 0 & 0\\ 0 & -0.5\tilde{G_{z}}I_{z} & 0\\ 0 & 0 & \tilde{G_{z}}I_{z} \end{array}\right] \end{array}$$ (7)

where *I_z_* is the current applied on the Maxwell coil, and $\tilde{G_{z}}$ is the derivation of the magnetic field in the z-direction on the z-axis when applying unit current. Without loss of generality, assume the magnetic moment direction of the magnet is *φ* in the x-y plane. Then, after applying current *I_z_* on the Maxwell coil, the force exerted on the magnet is derived by the following equation:

$$\begin{array}{c} \begin{array}{l} F_{m}=\left[\begin{array}{c} F_{x}\\ F_{y}\\ F_{z} \end{array}\right]\\ =\nabla B\cdot M\\ =\left[\begin{array}{ccc} -0.5\tilde{G_{z}}I_{z} & 0 & 0\\ 0 & -0.5\tilde{G_{z}}I_{z} & 0\\ 0 & 0 & \tilde{G_{z}}I_{z} \end{array}\right]\left[\begin{array}{c} M\;\text{cos}\;\varphi \\ M\;\text{sin}\;\varphi \\ 0 \end{array}\right]\\ =\left[\begin{array}{c} -0.5\tilde{G_{z}}I_{z}M\;\text{cos}\;\varphi \\ -0.5\tilde{G_{z}}I_{z}M\;\text{sin}\;\varphi \\ 0 \end{array}\right] \end{array} \end{array}$$ (8)

where *M* represents the magnitude of the magnetic dipole moment ***M***. It shows the direction of the propulsion force is always consistent with the direction of the magnetic dipole moment. Meanwhile, the magnitude of the magnetic force produced by the Maxwell coil is proportional to the current *I_z_*.

The microrobot is placed in a circular tank that is filled with silicone oil. The gravity and buoyancy balance each other in the vertical direction. As a result, we can focus on the forces acting in the x-y plane. The microrobot’s trajectory tracking control is divided into orientation and position control to ensure precision. For the orientation control, if the direction of ***B*** does not change too rapidly, it is reasonable to assume that ***M*** is always aligned with ***B*** [27]. According to our previous work [28], the direction of the permanent magnet experiences slight overshoot and oscillation and converges to the direction of the magnetic field in seconds. To eliminate unknown errors and reinforce the tracking ability, an inner proportional integral derivative (PID) controller is applied.

For the position control, the dynamic model is established as follows:

$$F_{m}+F_{\text{drag}}=ma$$ (9)

where ***F****_m_*, ***F****_drag_*, *m*, ***a*** denote the magnetic force, the viscous dragging force, the mass of microrobot and the acceleration of microrobot, respectively. Due to the low Reynolds number of the silicone oil, a linear relationship between the dragging force ***F****_drag_* and the microrobot’s velocity ***v*** exists [29], which is simplified as:

$$F_{\text{drag}}=-\;k_{d}v$$ (10)

where *k_d_* denotes the constant dragging coefficient. Substitute Eq. 10 into Eq. 9, then:

$$F_{m}-k_{d}v=ma$$ (11)

Because of the high viscous resistance of silicone oil, the microrobot will experience a brief acceleration, followed by a balance between magnetic and dragging forces, ultimately maintaining a constant speed of motion. So the relationship between the magnetic force and uniform speed in a steady state is expressed as follows:

$$F_{m}=k_{d}v$$ (12)

Substitute Eq. 8 into Eq. 12, then:

$$I_{z}=\frac{k_{d}}{0.5{\tilde{G}}_{z}M}v$$ (13)

where *v* represents the magnitude of velocity *v*. Therefore, the current of the Maxwell coil and the angular velocity of the magnetic field are controlled to decide the uniform speed and the angular velocity of the microrobot. The microrobot’s state **s** and input **u** are defined as:

$$\begin{array}{cc} s & ={\left[\begin{array}{cc} \begin{array}{cc} x & y \end{array} & \begin{array}{cc} v & \varphi \end{array} \end{array}\right]}^{T}\\ u & ={\left[\begin{array}{cc} I_{z} & \omega_{B} \end{array}\right]}^{T} \end{array}$$ (14)

where *x* and *y* are the 2-dimensional position, *φ* is the orientation, and *ω_B_* is the angular velocity of the uniform magnetic field. The state equation of the kinematic model can be calculated as:

$$s\left(t+1\right)=\mathrm{As}\left(t\right)+\mathrm{Cu}\left(t\right)+D$$ (15)

with

$$\begin{array}{cc} A & =\left[\begin{array}{cccc} 1 & 0 & \text{cosφ∆t}\; & -v\text{sinφ∆t}\;\\ 0 & 1 & \text{sinφ∆t}\; & -v\text{cosφ∆t}\;\\ 0 & 0 & 0 & 0\\ 0 & 0 & 0 & 1 \end{array}\right]\\ C & =\left[\begin{array}{cc} 0 & 0\\ 0 & 0\\ \frac{0.5{\tilde{G}}_{z}M}{k_{d}} & 0\\ 0 & \Delta t \end{array}\right]\\ D & =\left[\begin{array}{c} \mathrm{v\varphi}\text{sinφ∆t}\;\\ \mathrm{v\varphi}\text{cosφ∆t}\;\\ 0\\ 0 \end{array}\right] \end{array}$$ (16)

in which *Δt* is the sampling time of the discrete control system.

### System integration and control

**Fig. 5.**
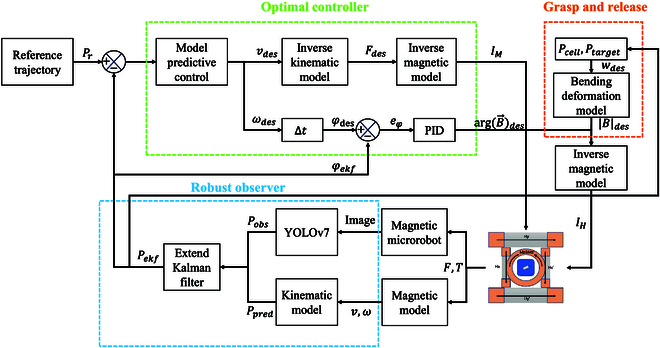
The schematic diagram of the control system. The robust observer for the position of the microrobot is applied to feedback precisely. The optimal controller is adopted for trajectory tracking. The proposed bending deformation model provides the basis for controlling the closing and opening of the microgripper.

In this paper, an EKF-MPC approach is designed to realize automatic trajectory tracking, and a bending deformation model is built for the grasping and releasing task. The schematic diagram of the proposed strategy is presented in Fig. 5. We first introduce EKF to fuse the kinematic predicted position and the observed position and obtain the robust positional estimation *P_ekf_*. Where the positional and directional feedback of the microrobot is achieved by YOLOv7 [30]. Next, an MPC is used to compute the control input for optimal path tracking. Then, the inverse model is applied to calculate the input of the Maxwell coil. At the same time, an inner PID controller is employed to control the orientation of the microrobot. A grasping and releasing module containing the proposed bending deformation model controls the magnitude of the uniform magnetic field, which results in the grasping and releasing motion of the microgripper. The overall system is designed to automate cell grasping and transportation operations.

The EKF fuses the pose predicted by the kinematic model with the pose observed by YOLOv7 to obtain more robust positional feedback. Considering situations with Gaussian errors in kinematic and observation models as follows:

$$\begin{array}{c} s\left(k\right)=p(s(k-1),u\left(k-1\right))+w\left(k-1\right),w\left(k-1\right)∼N(0,Q\left(k\right))\\ z\left(k\right)=h\left(s\left(k\right)\right)+g\left(k\right),g\left(k\right)∼N(0,R\left(k\right)) \end{array}$$ (17)

where ***s***(*k*), ***u***(*k*), ***w***(*k*), ***z***(*k*), ***g***(*k*), ***Q***(*k*), and ***R***(*k*) denote state vector, input vector, predictive noise vector, measured state, measured noise vector, predictive noise covariance matrix, and measured noise covariance matrix respectively at step *k*. Meanwhile, ***s***(*k*) = [*x*(*k*), *y*(*k*), *φ*(*k*)]*^T^*, ***u***(*k*) = [*I_z_*(*k*), *ω_B_*(*k*)]*^T^*, and *p* and *h* are kinematic model function and observation function. Firstly, the Taylor series expansion is applied to the nonlinear kinematic model and expressed by the Jacobian matrix:

$$\begin{array}{l} {\left.F=\frac{\partial p}{\partial s}\right|}_{\hat{s}\left(k-1\right),u\left(k-1\right)}\\ {\left.H=\frac{\partial h}{\partial s}\right|}_{\hat{s}-\left(k\right)} \end{array}$$ (18)

where ***F*** and ***H*** denote the Jacobian matrix of the kinematic model and observation model, respectively. The ${\hat{s}}^{-}\left(k\right)=$$p(\hat{s}(k-1),u\left(k-1\right))$ denotes the predicted state by the kinematic model. Meanwhile the $\hat{s}\left(k-1\right)$ in this equation denotes the last fused state. Then the optimal fusing state is calculated as follows:

$$\begin{array}{c} \begin{array}{cc} P^{-}\left(k\right) & \;=\mathrm{FP}\left(k-1\right)F^{T}+Q\\ K\left(k\right) & =P^{-}\left(k\right)H^{T}{\left({\mathrm{HP}}^{-}\left(k\right)H^{T}+R\right)}^{-1}\\ \hat{s}\left(k\right) & \;={\hat{s}}^{-}\left(k\right)+K\left(k\right)\left(z\left(k\right)-h\left({\hat{s}}^{-}\left(k\right)\right)\right)\\ P\left(k\right) & \;=\left(I-K\left(k\right)H\right)P^{-}\left(k\right) \end{array} \end{array}$$ (19)

where $P^{-}\left(k\right),K\left(k\right),\hat{s}\left(k\right)$, and ***P***(*k*) denote prior error covariance, Kalman gain, fused state, and updated error covariance, respectively.

The MPC solves optimal control inputs according to the kinematic model and constraints. It calculates multistep control to minimize the cost over horizons with the consideration of constraints and adopts the first optimal control in every step. The optimization problem is expressed as follows:

$$\begin{array}{c} \begin{array}{l} \underset{u}{\text{min}J}=\sum_{i=0}^{N}∥s-s_{\mathrm{ref}}{∥}_{Q}^{2}+\sum_{i=0}^{N-1}∥u-u_{\mathrm{ref}}{∥}_{R}^{2}\\ s.t.\;s\left(k\right)=\mathrm{As}\left(k-1\right)+\mathrm{Cu}\left(k-1\right)+D\\ \;u\left(k\right)\in \left[u_{\min},u_{\max}\right]\\ \;u\left(k\right)\in \left[u\left(k-1\right)-\Delta u_{\text{max}},u\left(k-1\right)+\Delta u_{\max}\right] \end{array} \end{array}$$ (20)

where *J* is the cost of MPC. *N* is the prediction horizon, ***Q*** and ***R*** are state and input weighting coefficient matrix. ***s****_ref_* = [*x_ref_*, *y_ref_*, *v_ref_*, *φ_ref_*]*^T^* and ***u****_ref_* = [*I_ref_*, *ω_ref_*]*^T^* are reference trajectory and input, respectively. ***u***_min_ and ***u***_max_ are lower and upper bound of input. *Δ****u***_max_ represents the maximum variation of input.

## Results

To verify the models mentioned above and the effectiveness of the proposed system, the microgripper bending deformation model verification, the trajectory tracking control, and cell grasping and transportation experiments are investigated.

### Microgripper calibration

**Fig. 6.**
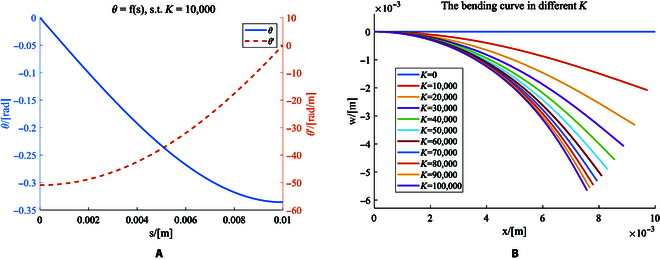
The numerical approximation of the bending deformation model. (A) The approximation of *θ* = *f*(*s*). (B) The numerical bending curve of the film in different magnetic fields.

There is no analytic solution for the nonlinear second-order differential equation in Eq. 6. Consequently, a numerical calculation is performed in MATLAB to investigate the mapping relationship *f*. In this study, *L* = 10 mm, *A* = *wh* = 1 mm^2^, $I=\frac{{\mathrm{hw}}^{3}}{12}=\frac{1}{48}$ mm^4^, *E* is a constant but difficult to measure. So $K=\frac{\mathrm{AB}}{\mathrm{EI}}$ is created to describe the variation of *B*. After approximation for *f* using the ode45 function in MATLAB as shown in Fig. 6A. *x(s)* and *w(s)* are calculated based on the integration that:

$$\begin{array}{cc} x\left(s_{0}\right) & =\int_{0}^{s_{0}}\;\text{cos}\left(f\left(s\right)\right)\mathrm{ds}\\ w\left(s_{0}\right) & =\int_{0}^{s_{0}}\;\text{sin}\left(f\left(s\right)\right)\mathrm{ds} \end{array}$$ (21)

Then the relationship between *x* and *w* under different *K* is illustrated in Fig. 6B. The degree of bending increases as the magnetic field increases, and the bending curve is infinitely close to the quarter circle when being magnetized.

**Fig. 7.**
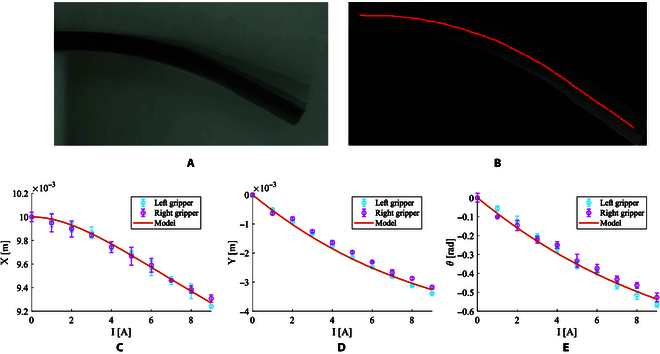
The verification of the proposed bending deformation model. (A) The superposition images of the static film under different magnetic fields. (B) The extraction for bending deformation curve from images. The approximation between the experimental data and model for the free endpoints of the 2 films in (D) *X*, (D) *Y*, and (E) *θ*.

To grasp cells precisely, the relationship between the deformation curve and the input current needs to be calibrated. In the real world, the 2 films used on the microgripper are actuated by the Helmholtz coil in the y-axis using a current of 0 to 9 A with intervals of 1 for 5 times. The images of the film are taken when its shape stabilizes under different magnetic fields as shown in Fig. 7A. After segmenting the film and approximating the deformation curve as shown in Fig. 7B, the position and orientation of the free endpoint are obtained. Finally, the linear coefficient between the current and *K* is approximated to fit the experimental data as shown in Fig. 7C to E. The fitting *R*^2^ of the *X*, *Y*, and *θ* in the left gripper are 0.996, 0.995, and 0.987. The fitting *R*^2^ of the *X*, *Y*, and *θ* in the right gripper are 0.990, 0.971, and 0.979. Although the bending of the right gripper is affected due to the attachment of a small piece of polydimethylsiloxane, compared with the fitted model, both the left and right grippers have shown a high level of consistency, indicating that the calibrated model can be effectively used to capture cells accurately.

### Magnetic trajectory tracking

**Fig. 8.**
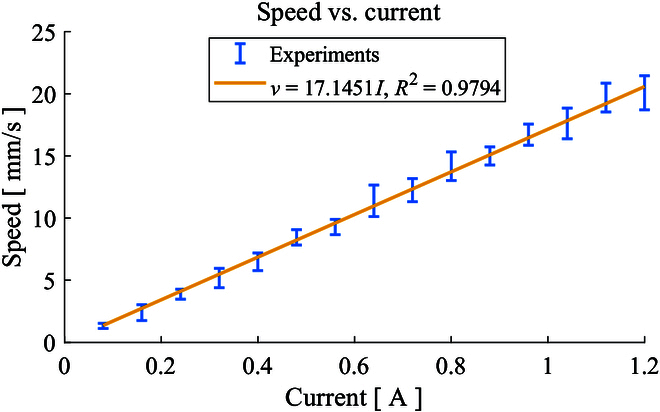
The relationship between the current in the Maxwell coil and the velocity of the microrobot.

To validate the linear relationship between the velocity of the microrobot with the current in the z-axis mentioned in Eq. 13, a kinematic model verification experiment is carried out. Figure 8 shows the 5 repetitive experiments and their linear approximation result which exhibit great linearity with a 0.9794 *R*^2^. The proportionality constant is also acquired and used to estimate the speed limit the microrobot can achieve which is approximately 30.86 mm/s under the maximum 1.8-A current.

**Fig. 9.**
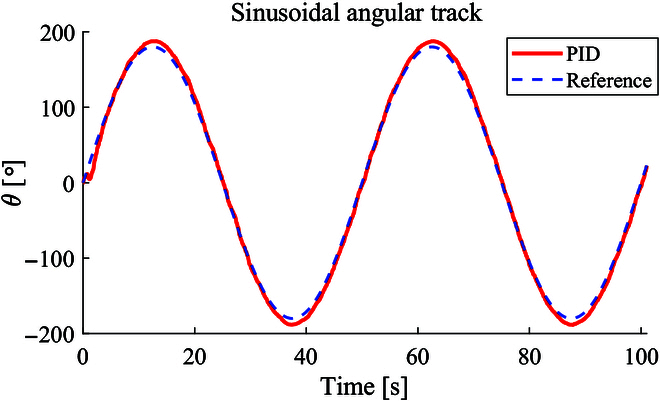
The PID angular control of the microrobot to track the sinusoidal reference.

Before path tracking, the angular tracking is evaluated. Because the angular error will influence the smoothness of the microrobot’s motion. In particular, when approaching the target cell, the angular control of the microrobot is important for grasping it. It is evaluated by a sinusoidal reference as shown in Fig. 9. The angular tracking is realized by a PID controller with *k_p_* = 0.03, *k_i_* = 0.10, *k_d_* = 0.01. The mean of absolute angular error is 3.57^∘^_,_ which is sufficient for the cell grasping and transportation task.

**Fig. 10.**
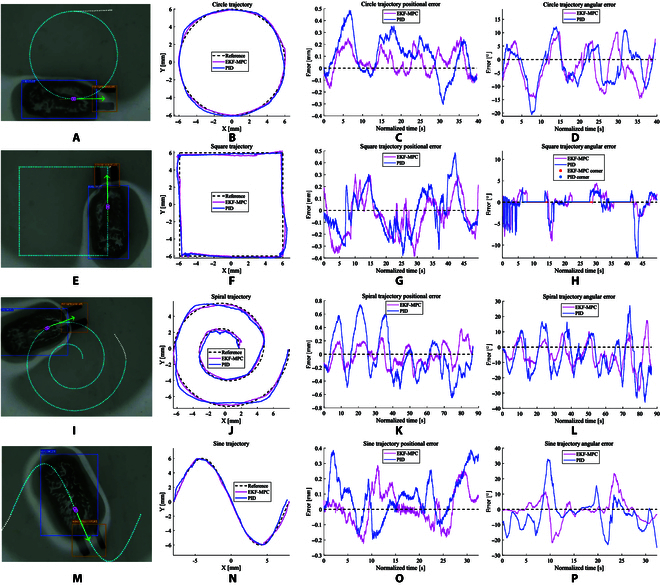
The closed-loop control of the microrobot to track circular, square, spiral, and sine trajectory. (A, E, I, and M) tracking process, (B, F, J, and N) tracking trajectory, (C, G, K, and O) positional error, and (D, H, L, and P) angular error.

To verify the effectiveness of the path following, circular, square, spiral, and sine reference trajectories are used. These references contain typical straight lines, turning points, and arc trajectories. To make the results more persuasive, the experiment was repeated 5 times for each trajectory and each method. Figure 10 shows the experimental results. Also, the tracking errors are listed in Table 2. The negative and positive positional errors are used to describe deviations from the reference trajectories. Negative errors indicate that the microrobot is deviating from the trajectory toward the inside, while positive errors indicate deviation toward the outside. MAE of the positional error is under 0.155 mm which is 1.55% of the total size of the microrobot.

**Table 2.** Error of trajectory tracking

Path	Method	Positional		Angular		Time (s)
		MAE (mm)	RMSE (mm)	MAE (^∘^)	RMSE (^∘^)	
Square	EKF-MPC	0.134±0.015	0.161±0.014	1.976±0.371	2.922±0.455	49.053±3.542
	PID	0.174±0.020	0.194±0.014	2.871±0.358	3.416±1.060	45.820±3.556
Circle	EKF-MPC	0.112±0.021	0.129±0.031	5.490±0.616	6.389±0.384	37.958±4.023
	PID	0.164±0.023	0.185±0.022	8.281±2.479	9.528±3.054	38.608±3.568
Spiral	EKF-MPC	0.155±0.017	0.166±0.012	9.660±0.656	9.829±0.788	75.531±9.867
	PID	0.267±0.028	0.294±0.040	12.165±0.946	12.406±1.165	73.985±8.516
Sine	EKF-MPC	0.094±0.004	0.110±0.005	6.329±0.381	8.838±0.533	32.302±0.884
	PID	0.130±0.005	0.163±0.028	7.600±0.390	10.031±0.655	32.543±1.149

### Cell grasping and transportation

#### Grasping characterization

The grasping reliability is investigated with the zebrafish embryonic cells in 5 different uniform magnetic fields. In each trial, a new zebrafish cell is placed at a random position in the container. Then, the zebrafish cell is grasped and moved 3 cm by the microrobot to observe whether the zebrafish cell is ejected from the microgripper during the movement. After each capture, the zebrafish cell is placed under a microscope to observe its integrity to determine whether it is damaged. Table 3 shows the results of 50 trials for each uniform magnetic field. With the increase of the magnetic field, the grasping force of the microgripper becomes stronger, thus improving the grasping success rate of the cells, but at the same time, the risk of cell damage is also increasing. In the actual experiment, a 3.0-mT uniform magnetic field is adopted for cell grasping, which not only ensures a high grasping success rate but also reduces the damage to the cells.

**Table 3.** Grasping characterization for zebrafish cells

Magnetic field (mT)	Success rate (%)	Damage rate (%)
2.0	52	0
2.5	74	0
3.0	94	0
3.5	98	2
4.0	100	6

#### Cell pick-and-place

**Fig. 11.**
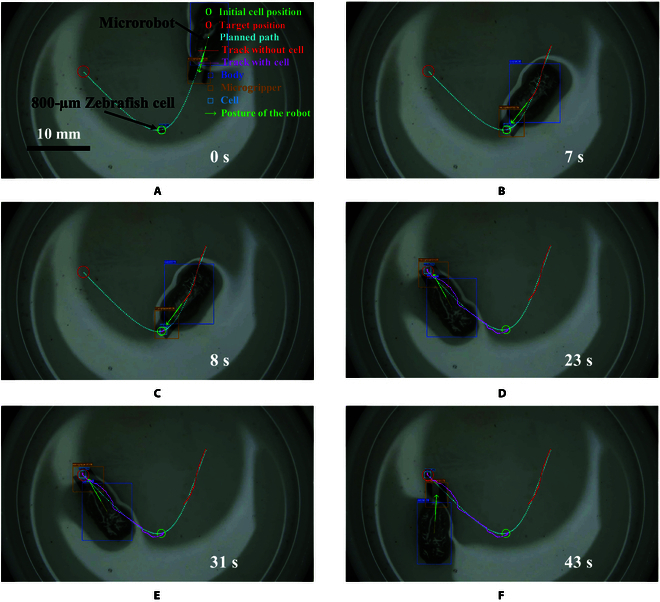
The process of grasping, transporting, and releasing a zebrafish embryonic cell using the proposed microrobot. (A) Initial state. (B) The microrobot approaches the zebrafish cell. (C) The microgripper closes to grasp the cell. (D) The microrobot transports the cell to the target location. (E) The microgripper opens to release the cell. (F) The microrobot goes back.

Five repetitive cell grasping and transportation experiments in liquid are investigated to confirm the effectiveness of this system. The red zebrafish embryonic cell (800-μm diameter) stained with Neutral Red Sterile Solution (G1310, Solarbio, China) is used to perform the task. The location of the zebrafish cell is detected by the YOLOv7 network. The target releasing place is determined by the mouse click in the microscope image graphical user interface. After detecting the initial position of the microrobot and the cell. A smooth path is planned through the Cubic Spline method using these 3 positions. The actions of grasping, moving, and releasing the zebrafish cell are illustrated in Fig. 11 with time stamps. Initially, the mobile microrobot approaches the target cell, as shown in Fig. 11A and B. Next, the microgripper closes according to the bending deformation model to grasp the cell as shown in Fig. 11C. Then, while grasping the cell, the microrobot moves to the target releasing place, as shown in Fig. 11C and D. Finally, the microgripper opens to release the cell, and the microrobot goes back, as shown in Fig. 11E and F. The average MAE of the microrobot trajectory tracking without the cell is 0.154 mm. After capturing the cell, the average MAE of the cell trajectory tracking is 0.215 mm. The mean cell release error for the 5 trials is 0.067 mm with a standard deviation of 0.013 mm.

## Discussion

The zebrafish embryo cell used in this study is relatively large compared with the common cells. The main challenge when working with smaller cells is ensuring precise grasping and manipulation due to the size difference. The microrobot’s gripping mechanism may need to be adapted or optimized to securely grasp smaller cells without damaging them. The image equipment and operation equipment for micro- and nano-scale is necessary to conduct experiments for smaller cells. It may also require more sophisticated control algorithms to accurately track and manipulate smaller cells in a more complicated environment. These challenges would need to be addressed through further research and optimization. In our current study, we primarily focused on suspension cells. However, the concept and techniques developed in our research can potentially be extended to work with adherent cells as well. Adherent cells present additional challenges due to their attachment to a substrate. To manipulate adherent cells, the microrobot would need to be equipped with appropriate tools or coatings to establish a gentle and controlled interaction with the cell without disrupting the cell–substrate adhesion. Innovative approaches, such as designing microscale grasping tools or utilizing gripping mechanisms with controlled grip strength, could be explored to address these challenges. Although the bending deformation model of the microgripper is effective for positional control, insufficient grasping force can easily lead to failure while excessive grasping force can damage the target cell. Hence, incorporating force feedback could be a viable solution in future work. The tracking error during the trajectory tracking tasks can be further optimized due to the variation of the rotational center of the microrobot. Therefore, an intelligent control algorithm for dealing with the time-varied system can be developed in the future.

In this study, a magnetic microrobot is proposed to grasp, transport, and release cells. A magnetic platform with minimal coils including 2 Helmholtz coils and 1 Maxwell coil is built to simultaneously control the motion and grasping of the microrobot. An EKF-based MPC is proposed to realize the path-tracking task accurately with an MAE of less than 0.155 mm. The bending deformation model of the microgripper is built and verified both in the numeric and real world and is applied to guide the closure of the microgripper precisely. Finally, a precise cell grasping and transportation task is completed with a releasing MAE of 0.067 mm to verify this system. This design provides a novel and feasible approach for cell manipulation research, with enormous potential for development.

## Data Availability

All data required to evaluate the conclusion of the paper is presented in this paper and supplementary materials. Additional data related to this article can be reasonably obtained from the authors.
